# Large Cohort Data Based Group or Community Disease Prevention Design Strategy: Strong Heart Study

**DOI:** 10.4236/wjcd.2018.83019

**Published:** 2018-03-27

**Authors:** Wenyu Wang, Elisa T. Lee, Barbara V. Howard, Richard Devereux, Ying Zhang, Julie A. Stoner

**Affiliations:** 1 College of Public Health, University of Oklahoma Health Sciences Center, Oklahoma City, OK, USA; 2 MedStar Health Research Institute, Hyattsville, MD, USA; 3 Weill Cornell Medical College, New York, NY, USA

**Keywords:** Group/Community Disease Prevention, Prevention Design, Prevention Strategy, Translate Study to Clinical Practice

## Abstract

**Background and Objective:**

A multitude of large cohort studies have data on incidence rates and predictors of various chronic diseases. However, approaches for utilization of these costly collected data and translation of these valuable results to inform and guide clinical disease prevention practice are not well developed. In this paper we proposed a novel conceptual group/community disease prevention design strategy based on large cohort study data.

**Methods and Results:**

The data from participants (n = 3516; 2056 women) aged 45 to 74 years and the diabetes risk prediction model from Strong Heart Study were used. The Strong Heart Study is a population-based cohort study of cardiovascular disease and its risk factors in American Indians. A conceptual group/community disease prevention design strategy based on large cohort data was initiated. The application of the proposed strategy for group diabetes prevention was illustrated.

**Discussion:**

The strategy may provide reasonable solutions to the prevention design issues. These issues include complex associations of a disease with its combined and correlated risk factors, individual differences, choosing intervention risk factors and setting their appropriate, attainable, gradual and adaptive goal levels for different subgroups, and assessing effectiveness of the prevention program.

**Conclusions:**

The strategy and methods shown in the illustration example can be analogously adopted and applied for other diseases preventions. The proposed strategy for a target group/community in a population provides a way to translate and apply epidemiological study results to clinical disease prevention practice.

## 1. Introduction

Prevention of chronic diseases has emerged as an urgent issue due to increasing prevalence of the chronic diseases and their effects on medical care, public health and economic burden. For example, it is estimated that >18 million Americans have diabetes (DM) and are at risk of related complications [1]. Several studies/trials have shown that DM may be prevented/delayed either through lifestyle or pharmacological interventions [2] [3] [4]. However, many important issues in designing an effective prevention program have not been considered or discussed sufficiently. These issues include complex associations of a disease with its combined and correlated risk factors, individual differences in health conditions, and selecting risk factors to target with interventions and setting appropriate treatment goals. On the other hand, large cohort studies have derived many results and collected datasets for risk factors of different diseases. Development of methods for utilization of these valuable results and costly data in designing more effective and efficient group/community disease prevention is still ongoing. In this paper, we proposed a conceptual group/community disease prevention design strategy based on data from large cohort studies, which might provide a way to translate and apply epidemiological study results to clinical disease prevention practice, and also reasonable solutions to the aforementioned issues. In this paper, we demonstrate how the proposed design strategy could be applied to prevent DM in American Indians (AI) based on the available data from the Strong Heart Study (SHS) [5]. The SHS is a population-based cohort study of cardiovascular disease (CVD) and its risk factors for American Indians in southwestern Oklahoma, central Arizona, and North and South Dakota.

## 2. Methods

Let us consider designing a disease prevention program to reduce, say, *m*% of incident risk of a disease in a given time period, say, four years, for a group/community (called the target group) in a population. We will show how to use an available disease risk prediction model and data from a large cohort study that includes the same or similar group (called the reference group) that are close to the target group in the prevention design.

### 2.1. Find a Cutoff Risk Level

To reduce risk of a disease for the target group through a prevention program, one intuitive way is to improve the profiles of risk factors of the disease in those individuals with high risk in the target group to the profiles in the others with not-high risk, and therefore to reduce overall incident risk of the disease for the target group. To implement this approach, we need to find a cutoff risk level (denoted as R*) to define and distinguish those with high risk in the target group, and then compare their risk profiles with that of the others with not-high risk in order to find which risk factors levels are needed to be improved through the prevention program. On the other hand, R* has to be defined in a way to approach our goal of reducing *m*% of incident risk of the disease in four years for the target group. We proposed the following way to find R*.

Let *RTG* denote the rate of the incidence risk of the disease in four years in the target group. If *RTG* is unknown, it can be estimated by the average of predicted disease risks (*APR*) in the reference group (*APRRG*) based on the risk prediction model of the disease and the risk factors data from the reference group. Let *r* denote a cutoff predicted risk (probability) of the disease, and *APRRG*(*r*) denote that the *APR* from those individuals with predicted disease risk <*r* in the reference group. Then, R* can be obtained from the following equation,

(1) $$R*=\max_{0<r<1}\left\{r,\mathrm{APRRG}\left(r\right)\leq “\mathrm{APRRG}\left(\text{or}\;\mathrm{RTG}\;\text{if}\;\text{it}\;\text{is}\;\text{known}\right)”-m\%\right\},$$

where *max* denotes the maximum. In practice, we can calculate *APRRG* (if *RTG* is unknown) first, then for each *r*, *r* = 0.01 to 0.99 by 0.01, calculate the respective *APRRG*(*r*) and check whether *APRRG(r)* ≤ “*APRRG*(or *RTG* if it is known)” − *m*%. Then R* is the largest such *r*. It is clear if through the prevention program, we can improve risk factors profiles of those individuals with predicted risk ≥R* to the profiles of those individuals with predicted disease risk <R* in the target group. Then, the overall *APR* from all individuals in the target group will be less than the *APR* from those individuals with predicted disease risk <R*, the latter is approximately equal to *APRRG*(R*). From (1), *APRRG*(R*) ≤ “*APRRG*(or *RTG* if it is known)” − *m*%, that is, we will expect to approach our goal of reducing the *m*% of incident risk for the target group.

A chronic disease is usually associated with many risk factors. For example, an incident DM is usually the result of combined effects of many risk factors such as fasting plasma glucose (FPG), hemoglobin A1c (HbA1c), waist circumference (WAIST), urinary albumin/creatinine ratio (UACR), and metabolic syndrome traits, and usually most of them are correlated [6] [7] [8] [9] [10]. Therefore, a prevention program focused on one or two risk factors may not be sufficient, and thus may decrease efficacy of the program. Furthermore, the usual way to set one uniform goal for a risk factor for all participants in a prevention program may not be appropriate or attainable due to individual differences in risk factors and health conditions, and sometimes may even cause adverse effects and safety problems. This may be one of the reasons that some clinical trials had to be discontinued in addition to medication toxicity problems. We proposed to conduct simultaneous intervention for all of the significant risk factors in the disease prediction model, and use the following methods to derive goal levels for each of the risk factors based on the data from the reference group.

### 2.2. Derive Goal Levels of All Risk Factors in the Disease Risk Prediction Model

To reduce effects of individual differences in risk factors and health conditions on setting goal levels for each of the risk factors, we divide all individuals in the reference group into subgroups based on some of the major risk factors in the prediction model, and derive goal levels for each of the risk factors separately for each of subgroups. Because the reference group is close to the target group, these derived goal levels of risk factors for each of subgroups based on the data from the reference group can be adopted as the respective goal levels for the respective subgroups of the target group. Prevention settings to achieve the goal levels of all risk factors for each prevention participant in the target group can then be designed individually based on his/her measured risk profile from the screening/baseline exam, respective subgroup goal levels, and prevention program. Individuals in each subgroup of the reference group will be classified as positive (if their “predicted incident risk from the prediction model” ≥ R*) or not-positive (otherwise). For each subgroup and a continuous risk factor, we propose to use a regression model to derive the goal for the risk factor. In the regression model, the risk factor is the dependent variable, and the other risk factors in the prediction model and a dummy variable (=1 if an individual is positive; =0, otherwise) are independent variables. Least-squares means (LSM) and 95% confidence interval (CI) of the risk factor for those positives and not-positives in the subgroup then can be estimated from the regression. The LSM represents the mean of the risk factor after adjusting for the other risk factors since they may be correlated. We propose to use the upper bound of the 95% CI of the LSM of the risk factor from those not-positives in the subgroup as the goal of the risk factor for the subgroup (the lower bound will be used if the risk factor is negatively associated with the disease in the prediction model). For a dichotomous risk factor, a similar procedure but a logistic regression will be applied. It is obvious that if the participants in each subgroup of the target group approach the goal levels of the risk factors for the subgroup through the prevention program, that is, their levels of risk factors will not differ significantly from those of not-positives, consequently their predicted disease risks will also approach the risk of those who are not positive (<R* as that in not-positives).

### 2.3. Assessments

Let *APR*_positives,_*_i_*, *APR*_not-positives,_*_I_* and *APR*_all,_*_i_* denote the average of predicted disease risks (APR) from those positives, not-positives and all in the *i*-th subgroup of the reference group, respectively. Then, the difference of *APR*_all,_*_i_* and *APR*_not-positives,_*_i_* can be used to predict prevention outcome for the *i*-th subgroup in the target group, and the difference of *APR*_positives,_*_i_* and *APR*_not-positives,_*_I_* can be used to predict prevention outcome for those positives. In addition, the weighted averages

(2) $$\sum_{i}n_{i}\mathrm{APR}\text{all},i/\sum_{i}n_{i}-\sum_{i}k_{i}\mathrm{APR}\text{not-positives},i/\sum_{i}k_{i}$$

(2a) $$\sum_{i}m_{i}\mathrm{APR}\text{positives},i/\sum_{i}m_{i}-\sum_{i}k_{i}\mathrm{APR}\text{not-positives},i/\sum_{i}k_{i}$$

where *n_i_*, *k_i_* and *m_i_* denote the number of all participants, those not-positives and those positives, respectively, in the respective *i*-th subgroup of the target group, will give the pre-assessed prevention outcome for the target group. The difference between APR based on the risk factors measurements at the screening/baseline exam for prevention and at the end exam of the prevention from each participant can be used as a score to estimate the true prevention effect.

## 3. Results

The following example illustrates how to apply the proposed disease prevention design strategy based on available results and data from a large cohort study.

### Illustration

Consider a DM (defined as having an FPG ≥ 126 mg/dl or HbA1c ≥ 6.5%) prevention to reduce 10% incidence risk of DM in four years for target group (aged 40+ years AI with a WAIST > 102 cm and free of DM).

### Available result

The following SHS DM risk (probability) prediction model [6] (or the respective DM risk-calculator at http://strongheart.ouhsc.edu).

(3) P(anindividualwilldevelopDMinfouryears)=1/(1+exp(−xbeta)),

where

(4) xbeta=11.3544−0.0292×Age+0.0167×WAIST+0.2856×I(elevatedbloodpresure)+0.0002×FPG×FPG−6.4798×HbAlc+0.6856×HbAlc×HbAlc+0.0192×Log(UACR)×Log(UACR)+0.3723×I(hypertriglyceridemia),

and in which the “elevated blood pressure” is defined as systolic blood pressure (SBP)/diastolic blood pressure(DBP) ≥ 130/85 mmHg or on hypertension (HTN) medication treatments, hypertriglyceridemia is defined as triglyceride (TG) ≥ 150 mg/dl, and *I*(.) the indicator function (for example, *I*(hypertriglyceridemia) = 1 if hypertriglyceridemia presented; = 0 otherwise).

### Available data

Data from the reference group (the SHS baseline (1989–1991) AI participants, aged 45 - 74, with WAIST > 102 cm and free of DM).

The characteristics for baseline participants of the SHS have been reported previously [11]. Based on Equation (1) and applying the methods explained in the subsection 2.1 of the Methods section, we have R* = 0.2945, which is solved based on the predicted DM risks from the SHS DM risk prediction model for the individuals in the reference group and the 10% reduction of incidence risk of DM in four years in the target group.

According to the methods explained in the subsection 2.2 of the Methods section, we divide all individuals in the reference group into four subgroups (FPG ≤ 106 mg/dL and HbA1c ≤ 5.3%, FPG ≤ 106 mg/dL and HbA1c 5.4% - 6.4%, FPG 107 - 125 mg/dL and HbA1c ≤ 5.3%, FPG 107 - 125 mg/dL and HbA1c 5.4% - 6.4 %) based on the 50th percentiles of FPG (106 mg/dl) and HbA1c (5.3%). Table 1 gives, for given R* = 0.2945 and the four subgroups, the information and the simultaneous goals (the bolded upper bound of 95% CI from those not-positive) for all risk factors in the DM risk prediction model.

For example, the regression model for deriving the upper bound of the 95% CI of the LSM of FPG from those not-positives in a subgroup (the goal level of risk factor FPG for the subgroup) is as follows.

(5) FPG=b0+b1×I(individualispositive)+b2×Age+b3×WAIST+b4×I(HTNmedications)+b5×SBP+b6×DBP+b7+HbAlc+b8×Log(UACR)+b9×Log(TG)+ε

where *ε* denotes the error term and *I*(.) is the indicator function.

To use Table 1 in the DM prevention, say, at the screening exam, those AI in the target group, who would be identified as belonging to the last subgroup (FPG in 107 - 125 mg/dl and 5.3% < HbA1c < 6.5%) in Table 1, should reduce/keep their FPG, HbA1c, UACR, TG and WAIST levels below the goal levels of 112 mg/dl, 5.6%, 6 mg/g, 125 mg/dl and 113 cm, respectively; and SBP/DBP below 129/77 mmHg if not on HTN medication treatments to prevent incident DM. The reductions in TG and SBP/DBP are also implied the participants in this subgroup should not have either elevated TG or elevated blood pressures, or should reduce their rates of elevated TG and elevated blood pressures below the goal rates of 13.2% and 51.9% (Table 1), respectively, to prevent incident DM. If the participants in this subgroup all approach/keep the goal levels, then it is expected that their risk of incident DM will be reduced to 24.9% (APR = 24.9% for not-positives in this subgroup) from 41.3% (APR = 0.413 for the whole subgroup), in which, those positives will reduce more from APR = 45.6% (APR = 0.456 for positives in this subgroup).

**Figure 1:**
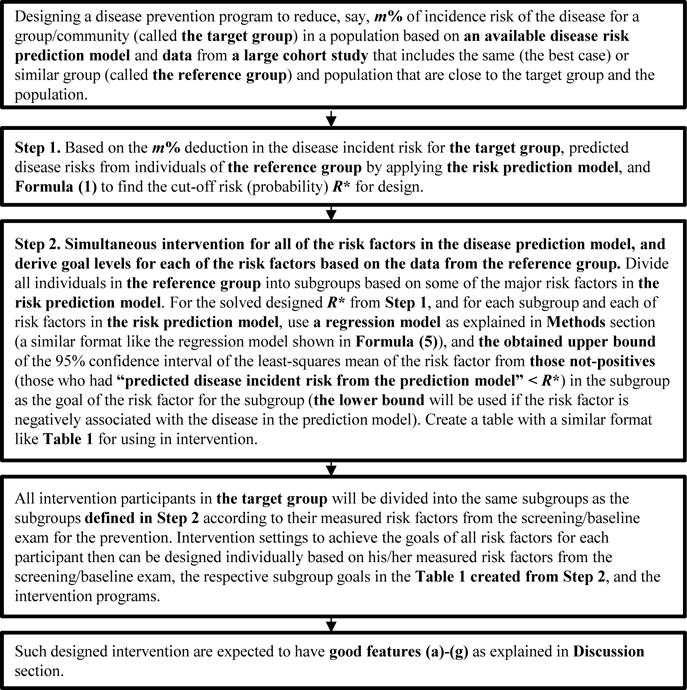
Design strategy for a group/community disease prevention based on large cohort data.

Figure 1 provides a summary of the proposed design and strategy.

## 4. Discussion

A chronic disease is usually the result of combined effects of many risk factors in which most of them are correlated. We propose to use the available disease risk prediction model in the design and assessments of the prevention effects since the predicted risk represents optimal combined risk and correlated effects of major risk factors of the disease. Recent clinical trials demonstrate that, for incidence, lifestyle/pharmaceutical interventions may prevent development of DM [12] [13] [14]. However, the question of how a DM prevention should be monitored is not clear [15]. Compared with the usual way of setting uniform goals for one/two risk factors for all participants in an intervention, we proposed to conduct simultaneous intervention for all significant risk factors in the disease risk prediction model, and proposed a way to set goal levels for the all risk factors and vary them for different subgroups. Our proposed strategy has the following features.

- Addressed complex associations of a disease with its combined and correlated major risk factors, and used all available valuable results and costly collected data in the design.
- It is considerable that individuals in the same subgroup have approximately similar health conditions. The proposed goal levels based on the levels of risk factors from those not-positives in the same subgroup accommodate subgroup differences and the combined and correlated effects of the disease risk factors. Therefore, these proposed goal levels might be more appropriate, attainable and safe compared to those usual way of setting a uniform goal level for all participants in an intervention. Moreover, in an intervention, for a participant in a subgroup, if his/her levels of some risk factors are already satisfying the respective goals, no interventions for these risk factors will be conducted, and thus is cost-saving.
- The derived information and goal levels (e.g. Table 1) can be used for the awareness of the disease, risk factors of the disease, and intervention effects for health providers and participants. For example, in the last subgroup in Table 1, the LSM of FPG, HbA1c, UACR and WAIST, the hypertriglyceridemia and elevated-blood-pressure rates between positives and not-positives were significantly different (all p < 0.002; all significant p-values were bolded in Table 1). Thus these risk factors are the reasons why some individuals in this subgroup were positive while the others were not, and thus should get more attention in intervention. Moreover, the estimated average predicted probabilities (APR) of the disease for positives and not-positives in different subgroups based on the data from the reference group can also be used to show potential intervention benefits. For example, for positives in the target group who belong to the last subgroup in Table 1, their APR might be 45.6% if without intervention. However, if they approach all their goal levels through the intervention, their APR might be reduced to 24.9% (the level of those not-positives).
- Table 1 shows a suggestion for a gradual intervention. For example, the 4th and 3rd subgroups were defined by the same FPG range but different HbA1c ranges, and the goals for HbA1c were gradually relaxed from <5.6% to <5.0%. Therefore, in intervention, an individual belonged to the 4th subgroup would be instructed to reduce/keep his/her level of HbA1c to <5.6%, while the 3rd subgroup <5.0%. Of course, participants in the 4th subgroup would not be discouraged to reduce their level of HbA1c to <5.0% (the goal for the 3rd subgroup), but they could do this gradually (first <5.6 then <5.0%) and thus more safe and attainable. This feature may reduce frustrations of participants who have more serious health conditions but be stressed to rapidly reduce their risk factors levels to common goals for everyone in an intervention. This feature may be also necessary considering a chronic disease is a timely cumulative outcome of combined risk factors, and therefore the return to normal levels of the risk factors should be also a timely and gradual procedure.
- Table 1 shows also an adaptive strategy for the intervention. For example, if an individual belongs to the last subgroup (FPG in 107 - 125 mg/dl and 5.3% < HbA1c < 6.5%) at the beginning of the intervention and his/her HbA1c is later reduced to ≤ 5.3% while FPG remained unchanged during the intervention, and the improved HbA1c remains stable in perhaps two consecutive visits, then his/her goal levels and intervention settings could be adaptively changed to those in the 3rd subgroup with FPG in 107 - 125 mg/dl and HbA1c ≤ 5.3%.
- Easy prediction and assessments for the intervention as explained in Methods section.
- Learnable. Data collected from the intervention may be added to the already collected data, and the expanded data then may be used to improve/update the disease-predictive model and the subgroup goals for the future intervention.

We proposed and demonstrated how to utilize and translate the available research results and data in designing a group/community disease prevention program, and assess/predict the effectiveness of the program. The strategy and methods shown in the illustration example for DM prevention can be analogously adopted and applied for other diseases preventions. To our knowledge, the proposed design strategy is new the first time in its kind which represents a novel frame work for the utilization and translation of large collected data. However, such design strategies need to be tested and validated in real disease prevention studies. The proposed strategy depends on a disease-predictive model and risk factors data from the same population of the target group. If the needed information is not available from the same population, one may use available information from another population that closely resembles the population under study. Only four subgroups were demonstrated in Table 1 due to the limited sample size. We may expect the learnable feature g) of our strategy will allow us to define more subgroups and thus set more appropriately individualized goals in the future.

## 5. Conclusion

The proposed design strategy considers the complex associations of a disease with its combined and correlated risk factors and individual differences; provides ways to simultaneously set gradual, attainable and safe goals for all risk factors in different subgroups; and forms an adaptive intervention frame. The proposed design strategy represents a way to utilize or translate available valuable results and costly collected data from large cohort studies for clinical disease prevention practice, and can be applied for group/community diseases preventions.

## Figures and Tables

**Table 1:** For designed cutoff probability R* = 0.2945, suggested intervention goals (bolded upper bound of 95% CI) for DM risk factors for four subgroups.

FPG	HbA1c	Not-Positive				

(mg/dl)	(%)	Risk Factor	LSM	95% CI		*P*^a^
≤106	≤5.3	**FPG (mg/dl)**	97	96	**98**	0.06
		**HbA1c (%)**	4.9	4.8	**4.9**	**0.02**
**Not-Positive**^d^	**Positive**^d^	**UACR (mg/g)**	6	5	**7**	<**0.001**
n = 257	n = 21	**TG (mg/dl)**	113	107	**120**	**0.002**
APR = 0.164	APR = 0.356	**TG ≥ 150 mg/dl**	21.70%	16.81%	**27.48%**	<**0.001**^c^
APR-all =	0.178	**SBP/DBP ≥ 130/85 mmHg or on medication for HTN**	59.70%	53.16%	**65.98%**	**0.02**^c^
		**DBP**^b^ **(mmHg)**	77	76	**78**	**0.004**
		**SBP**^b^ **(mmHg)**	123	121	**124**	**0.003**
		**WAIST (cm)**	112	111	**113**	<**0.001**

≤106	5.4 - 6.4	**FPG (mg/dl)**	97	96	**98**	<**0.001**
		**HbA1c (%)**	5.6	5.6	**5.7**	<**0.001**
**Not-Positive**	**Positive**	**UACR (mg/g)**	7	5	**10**	0.06
n = 79	n = 69	**TG (mg/dl)**	117	105	**129**	0.13
APR = 0.210	APR = 0.405	**TG ≥ 150mg/dl**	13.30%	6.57%	**25.02%**	**0.008**
APR-all =	0.301	**SBP/DBP ≥ 130/85 mmHg or on medication for HTN**	46.90%	30.94%	**63.44%**	**0.005**
		**DBP (mmHg)**	74	72	**76**	**0.03**
		**SBP (mmHg)**	122	119	**125**	0.07
		**WAIST (cm)**	112	110	**115**	<**0.001**

107 - 125	≤5.3	**FPG (mg/dl)**	112	111	**113**	<**0.001**
		**HbA1c (%)**	4.9	4.9	**5.0**	0.3
**Not-Positive**	**Positive**	**UACR (mg/g)**	6	5	**8**	**0.03**
n = 114	n = 63	**TG (mg/dl)**	115	106	**125**	<**0.001**
APR = 0.218	APR = 0.360	**TG ≥ 150 mg/dl**	7.70%	3.79%	**14.98%**	<**0.001**
APR-all = 0.268		**SBP/DBP ≥ 130/85 mmHg or on medication for HTN**	35.80%	25.22%	**48.04%**	<**0.001**
		**DBP (mmHg)**	75	74	**77**	0.05
		**SBP (mmHg)**	120	118	**123**	**0.001**
		**WAIST (cm)**	111	110	**113**	<**0.001**

107 - 125	5.4 - 6.4	**FPG (mg/dl)**	111	109	**112**	<**0.001**
		**HbA1c (%)**	5.6	5.5	**5.6**	<**0.001**
**Not-Positive**	**Positive**	**UACR (mg/g)**	3	2	**6**	**0.002**
n = 39	n = 151	**TG (mg/dl)**	108	93	**125**	0.09
APR = 0.249	APR = 0.456	**TG ≥ 150 mg/dl**	4.20%	1.22%	**13.22%**	<**0.001**
APR-all = 0.413		**SBP/DBP ≥ 130/85mmHg or on medication for HTN**	32.20%	17.27%	**51.96%**	**0.002**
		**DBP (mmHg)**	75	72	**77**	0.64
		**SBP (mmHg)**	125	121	**129**	0.85
		**WAIST (cm)**	111	108	**113**	<**0.001**

a p-value from testing the difference of least-square means between positive and not-positive AI in a subgroup.

b The results for DBP and SBP are based on data from those without hypertension medications treatments.

c p-value from testing the difference of least-square rates of the metabolic syndrome trait between positive and not-positive AI in a subgroup based on a logistic regression model.

d Individuals in each subgroup of the reference group will be classified as positive (if their “predicted incident risk from the prediction model” ≥ R*) or not-positive (otherwise). AI, American Indians; CI, confidence interval; DBP, diastolic blood pressure; n, the sample size; APR, average predicted probability of developing DM in four years; APR-all, APR from all individuals in the subgroup; FPG, fasting plasma glucose; HbA1c, hemoglobin A1c; HTN, hypertension; LSM, least-square mean; SBP, systolic blood pressure; TG, triglycerides; UACR, urinary albumin and creatinine ratio; WAIST, waist circumference. SI conversions: to convert TG to mmol/L, multiply by 0.0113; covert FPG to mmol/L, multiply by 0.0555.
